# Medicinal plants species used by herbalists in the treatment of snakebite envenomation in Uganda

**DOI:** 10.1186/s41182-020-00229-4

**Published:** 2020-06-05

**Authors:** David Fred Okot, Godwin Anywar, Jane Namukobe, Robert Byamukama

**Affiliations:** 1 Department of Chemistry, Makerere University, P. O. Box 7062, Kampala, Uganda; 2 Department of Chemistry, Gulu University, P. O. Box 166, Gulu, Uganda; 3 Department of Plant Sciences, Microbiology & Biotechnology, Makerere University, P.O. Box 7062, Kampala, Uganda

**Keywords:** Medicinal plants, Envenomation, Snakebite, Traditional medicine practitioners, Post-conflict, Uganda

## Abstract

**Background:**

There are high mortality and morbidity rates due to poisonous snakebites globally with sub-Saharan Africa having some of the highest cases. However, traditional medicine practitioners (TMP) have been treating snakebites in Uganda for long despite the fact that few studies have been conducted to document such vital and rich indigenous traditional knowledge before it is lost. This study aimed to document the medicinal plant species used by experienced TMP in treating snakebite envenomation in selected post-conflict parts of Uganda. An ethnopharmacological survey was conducted in Kitgum, Serere, Kaberamaido and Kaabong districts in Uganda. Twenty-seven TMP with expertise in treating snakebites were purposively identified using the snowball technique and interviewed using semi-structured questionnaires. Data were analysed using simple descriptive statistics.

**Results:**

Sixty plant species from 28 families were documented with high consensus among the isolated indigenous Ik tribe of Kaabong district. Most of the plant species used were from the Asteraceae and Fabaceae families with eight species each. The genus *Echinops* was the most well-represented with three species*.* The most commonly used plant species were of citation were *Steganotaenia araliaceae* (16), *Microglossa pyrifolia* (Lam.), *Gladiolus dalenii* Van Geel (13), *Aframomum mildbraedii* Loes. (11), *Jasminum schimperi* Vatke and *Cyathula uncinulata* (Schrad) Schinz (10) and *Crinum macowanii* Baker and *Cyphostemma cyphopetalum* (Fresen.) Desc. ex Wild & R.B. Drumm (10)*. S. araliaceae* which was mentioned by all the TMP in the Ik community was used for first aid. Most of the plant species were harvested from the wild (68.75%) and were herbs (65.0%) followed by trees (23.3%). The most commonly used plant parts were roots (42.6%) and leaves (25.0%). Thirteen different methods of preparation and administration were used. Most of the medicines were administered orally (61.2%) and topically (37.6%). The commonest methods of oral application were cold water infusions (32.5%) and decoctions (21.7%).

**Conclusions:**

TMP widely use several medicinal plant species for treating snakebite envenomation in the selected post-conflict regions of Uganda

## Background

Worldwide, more than five million people suffer snakebite envenomation leading to 25,000–125,000 deaths, while an estimated 400,000 people are left with permanent disabilities [1]. The World Health Organization (WHO) has classified snakebites as one of the most neglected tropical diseases (NTD) in terms of incidence, severity, and clinical characteristics. This has served as a basis for the advocacy for snakebite envenomation [2]. The burden of snakebite envenomation was eventually recognized in June 2017 and then enlisted as a NTD category A by WHO [2, 3].

Snakebite envenoming primarily affects residents of rural communities in Africa, Asia, Latin America and New Guinea and possess a serious health challenge [4]. It is an occupational, environmental and domestic health hazard that exacerbates the already impoverished state of these communities [1]. Venoms consist of mainly toxic modified saliva of poisonous snakes. They are complex mixtures of enzymes, proteins, non-proteins and metalloproteinases [5]. The most important venom components that cause serious clinical effects are pro-coagulant enzymes, cytolytic or necrotic toxins, haemolytic and myolytic phospholipases A2, pre-synaptic and post-synaptic neurotoxins and haemorrhagins [6]. Broadly, there are two types of toxins, namely, neurotoxins, which attack the central nervous system, and haemotoxins, which target the circulatory system and kill victims very first. Snakes with neurotoxic venom include cobras, mambas, sea snakes, kraits and coral snakes [5]. Snakes with haemotoxic venom include rattlesnakes, copper head and cottonmouths [7]. Snake venoms can be neutralized by antibodies obtained after immunizing domestic animals with them. This led to the production of anti-venom called antisera. A major drawback of serum therapy is its prohibitive cost and chance that victims are often some distance away from medical care when bitten [8]. The search for novel venom inhibitors from natural products is therefore relevant because of their potential to complement serum therapy in neutralizing mainly the local damages of envenomation. Plant extracts constitute an excellent alternative with a range of anti-venom activities [7].

Africans have traditionally been treating poisonous snakebites using herbs [9–12]. For instance, of the 147 patients bitten by snakes seen between November 1995 and October 1996, 90% of them used herbs in KwaZulu-Natal, South Africa [13]. In Kenya, 32 medicinal plants have been documented for the treatment of snakebites [14, 15]. In central Uganda, 36 plant species were documented for treating snakebites [10]. A total of 25 plants were documented for the treatment of snakebites in eastern Uganda, Bulamogi County, Uganda [11]. Five other medicinal plant species were documented for treatment of snakebites in the Northern sector of Kibale National Park in western Uganda [16].

The current population of Uganda is over 45.3 million [17]. More than 80% of Ugandans are involved in agriculture and live in rural areas [18]. This makes them highly vulnerable to snakebite envenomation coupled with lack of access to antisera in health facilities. There is a widespread use of medicinal plants for the treatment of snakebites in Uganda. However, there are no statistics available on snakebite envenomation and treatment. Additionally, ethnopharmacological surveys of plants used for the treatment of snakebites have not been done in many parts of Uganda. This study aimed to document the plant species used in the treatment of snakebite envenomation in the Acholi, Teso and Karamoja sub-regions of Uganda. These are post-conflict regions and were affected during the war led by Joseph Kony’s Lord’s resistance army (LRA) rebel outfit. The LRA war begun in 1986 and lasted over 18 years [19]. Anecdotal evidence points to a high prevalence of snakebite envenomation experienced by returnees during the post-conflict resettlement in northern Uganda. This is because as many as 2 million people who had fled the fighting were forced into internally displaced people’s camps in north of the country [20].

## Results

Twenty-seven TMP were purposively selected and interviewed. Twenty-five were women and the rest were men. The average age of the respondents was 54.7 years and ranged from 36 to 95 years. The majority of the respondents (80%) were illiterate, with only 20% having attained primary education and were all peasant farmers (Table 1).

**Table 1:** Socio demographic characteristics of the traditional medicine practitioners

TMPS interviewed	
Age range	36–95
Females	5
Males	22
**Total**	27
**Level of education**	
Diploma	0
Advanced level	0
Ordinary level	0
Primary	12
None	15

**Fig. 1:**
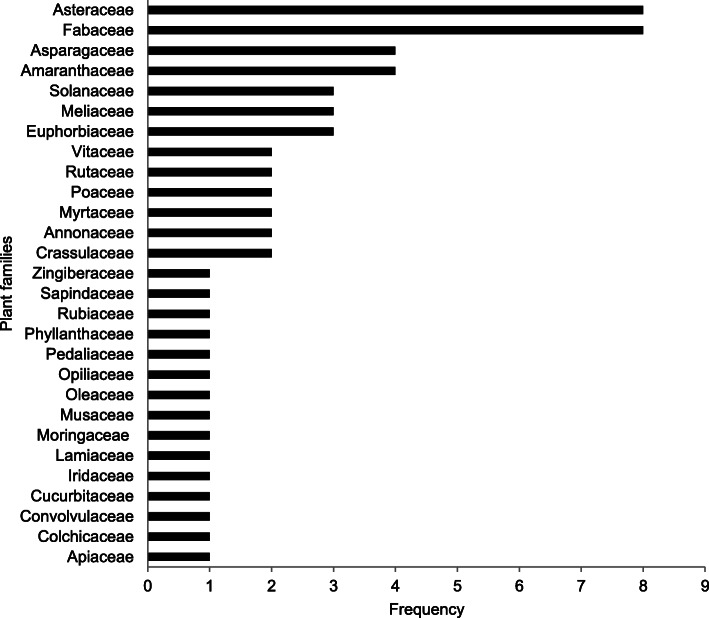
Families of medicinal plant species used in the management of snakebite envenomation in the Acholi, Teso and Karamoja sub-regions of Uganda

Sixty plant species from 28 families and fifty-one genera were documented (Table 2). The plant families with most species were Asteraceae (8), Fabaceae (7), Asparagaceae and Amaranthaceae with 4 species each and Euphorbiaceae, Meliaceae and Solanaceae with 3 species each (Fig. 1). The genus with the most plant species was *Echinops* (3). This was followed by *Annona*, *Chlorophytum* spp., *Eucalyptus* and *Solanum* with two species each (Table 2).

**Table 2:** Medicinal plant species used in the management of snakebites in Acholi, Teso and Karamoja sub-regions of Uganda

Family/scientific name (voucher number)	Local name (language)	Parts used	Habit	Mode of preparation and administration	Wild/domesticated	Frequency of mention	Documented use in the treatment of snakebite envenomation elsewhere
**Amaranthaceae**							
1.* Cyathula uncinulata* (Schrad) Schinz (ODF 001)	Kulabakak (Ik)	R	H	Apply powder to bite area after making small cuts with a razor blade.	W	10	No reports
**Amaryllidiaceae**							
2. *Allium cepa* L. (ODF 019)	Tungulu (Luo)	Blb	H	Decoction and drink	D	1	Externally applied for the treatment of snakebite in Salem district of India [21] and Colombia [22]. Bulbs are chewed for snakebite in eastern and central Uganda [10, 11].
3.* Ammocharis tinneana* (Kotschy & Peyr.) Milne-Redh. & Schweick (ODF 025)	Joda (Luo)	L	H	Decoction and drink	D	1	No reports
4. *Crinum macowanii* Baker (ODF 20)	(Ateso)	B	H	Powdered and mixed with powder of *C. cyphopetalum* and applied topically. Powder also dissolved in and drink.		10	No reports
**Annonaceae**							
5. *Annona chrysopylla* (ODF 023)	Obolo (Luo)	L, R St	Sh	Decoction. Stems and leaves used for repelling snakes	W	4	No reports
6. *Annona senegalensis* Pers. (ODF 002)	Obolo (Luo)	R/L	T	Pound and mix with water. Drink once/chew root and apply on the bitten area the next day. Stems barks used to repel snakes	W/D	9	Methanolic leaf extracts inhibited *Echis ocellatus* (Viper) venom activities [23]. Methanol root extract reduced hyperthermia and directly detoxified snake venom by 16–33% in rats against cobra (*Naja nigricotlis nigricotlis*) venom in rats [24].
**Apiaceae**							
7. *Steganotaenia araliaceae* Hochst. (ODF 003)	Segere (Ik)	L	S	Chew and swallow juice as first aid. Pound leaves, mix with water & wash out the venom from eyes to avert blindness.	W	16	Used in western Kenya for snakebite [14]
**Asparagaceae**							
8. *Albuca abyssinica* Jacq. (ODF 004)	Amujej (Ateso)	Blb/L	H	Crush leaves/bulbs, mix with water and drink as a purgative/apply on the bitten area/planted as a snake repellent	W	3	No reports
9. *Chlorophytum* spp 1 (ODF 022)	Emutungulu akwangan (Ateso)	Tb	H	Pound and apply on the snake bitten area	D	2	No reports
10. *Chlorophytum* spp 2 (ODF 024)	Eryau (Ateso)	Tb	H	Chew fresh roots	D	2	No reports
11. *Sansevieria trifasciata* Prain (ODF 036)	Tworo (Luo)	L	H	Pound and drink juice. Apply topically	W	3	Snake bites and poison antidote in southern Uganda [25]
**Asteraceae**							
12. *Echinops longifolius* A. Rich. (ODF 011)	Ofilifil (Ik), okeya (Luo)	L	H	Burn to make and apply on bitten site once only/rub directly on bitten part/mix 1 tsp with water.	W	9	No reports
13. *Echinops amplexicaulis* Oliv. (ODF 013)	Lukwang (Luo)	R	H	Pound, mix with water and drink once only/chew and apply on site the next day	W	3	Used in northern Uganda [26]. A novel crystalline caffeic acid from roots has anti-venom agents for hemolytic snake venoms [27].
14.* Echinops issphaerocephalu*s L (ODF 005)	Okeya (Luo)	R	H	Pound, mix with water and drink once only/chew and apply on site the next day	W	2	No reports
15.* Erigeron floribundus* (Kunth) Sch.Bip. (ODF 021)	Ejut dolei (Ateso)	L	H	Squeeze juice and drink 3 times a day for at least 3 days	W	3	No reports
16. *Lactuca inermis* Forssk. (ODF 027)	Ekile (Ateso)	R	H	Mix the powder with cold water & drink 3 times a day for at least 3 days	W	3	No reports
17.* Microglossa pyrifolia* (Lam.) Kuntze (ODF 006)	Ekiya Lo’emun (Ik), Etutum (Ateso)	R	H	Pound and mix with water and drink for 2 days/mix powder with cold water and drink 3 times a day for at least 3 days	W	13	Used in Mukono district in central Uganda for snakebite treatment [10, 28]. An infusion is drunk for snakebite [11].
18. *Sigesbeckia orientalis* L. (ODF 035)	Yat twol (Luo)	L	H	Squeeze juice and drink/paste apply topically	W	5	No reports
*19. Vernonia biafrae* Oliv. & Heirn (ODF 030)	Ebwolibwol (Ateso)	R	H	Pound and mix with water and drink as a purgative	W	2	No reports
**Colchicaceae**							
20. *Gloriosa superba* L (ODF 007)	Lobon bong (Ik)	R	H	Powder sometimes mixed with the powder of *G. dalenii* for various snake types, spider and scorpion stings.	W/D	8	No reports
**Convolvulaceae**							
21.* Astripomoe amalvacea* (Klotzsch) A. Meeuse (ODF 008)	Apom (Ateso)	R/St	H	Pound and mix with water and drink once a day for 2–5 day	W	3	A paste from the tuber is applied externally on snakebite wounds [21, 29].
**Crassulaceae**							
22. *Kalanchoe* sp. (ODF 032)	Ecucuka (Ateso)	L	H	Leaf juice/paste taken orally	W/D	2	No reports
23.* Bryophyllum delagoense* (Eckl. & Zeyh.) Druce (ODF 031)	Omucaga (Ateso)	L	H	Leaf juice/paste taken orally	D	2	No reports
**Cucurbitaceae**							
24. *Coccinia grandis* (L.) Voigt (ODF 033)	Bomo twol (Luo)	R	H	Decoction	W	2	No reports
**Euphorbiaceae**							
25.* Euphorbia hirta* L (ODF 029)	Acakacak (Luo) Orurungo (Ateso)	B	H	Decoction	W	5	Roots are eaten for snakebite in central Uganda [10].
26. *Euphorbia hypericifolia* L. (ODF 009)	Loje (Ik)	L	H	Pound/squeeze juice and apply directly to bitten part twice a day for 2 days	W	6	No reports
27. *Euphorbia tirucalli* L. (ODF 028)	Kilajok (Luo)	Sp	H	Drink sap and apply topically	D	2	Western Uganda [16]
**Fabaceae**							
28. *Canavalia ensiformis* (L) DC. (ODF 026)	Yat twol (Luo)	Sd	H	Chew seeds	D	2	No reports
*29. Glycine max*(L.) Merr. (ODF 037)	Soya (Luo)	Sd	H	Chew seeds	D	1	Seeds used in central Uganda [10].
30. *Indigofera arrecta* A.Rich. (ODF 040)	Eragwii (Ateso)	R		Decoction and drink or powder applied topically	W	5	Roots used for snakebites as a poultice [11]. A leaf infusion drunk for snakebites [30]
31. *Indigofera spicata* Forssk. (ODF 038)	Yat twol (Luo)	R, L and S	H	Pound and drink juice and apply topically	W	8	No reports
32. *Lonchocarpus laxiflorus* Guill. & Perr (ODF 059)	Eputon (Ateso)	R	T	Vomiting	W	1	No reports
33. *Piliostigma malabaricum* (Roxb.) Benth. (ODF 046)	Ogali (Luo)	T	L/B	Decoction	W	2	No reports
34. *Tamarindus indica* L.	Chwaa (Luo)	T	Sd	Chew/apply to the snake-bitten area	W/D	7	Seeds are crushed & taken orally as anti-venom [31]. Seeds used for scorpion stings [32]
*35. Senna hirsuta* (L.) H.S.Irwin & Barneby (ODF 050)	Elekumare (Ateso)	R	T	Mix the powder with cold water and drink 3 times daily for at least 3 days	W	3	No reports
**Iridaceae**							
36. *Gladiolus dalenii* Van Geel (ODF 014)	Lodokole (Ik)	B	H	Make small cuts around the bitten area & apply powder once/mix powder with water & drink	W	13	Venomous stings & bites in Cameroon [33].
**Lamiaceae**							
37. *Hoslundia opposita* Vahl (ODF 010)	Etutu/Tutu (Ateso) Itutu (Kumam)	R	Sh	Crush in water and drink/rub on bitten part/powder and mix with about ¼ l of warm water and drink twice a day for 3 days	W	5	Root chewed and make a poultice for snakebites [11].
**Meliaceae**							
38. *Azadirachta indica* A.Juss. (ODF 039)	Neem* (Acholi)	F	T	Decoction	D/W	6	A decoction or poultice used in central Uganda [10].
*39. Pseudocedrela kotschyi* (Schweinf.) Harms (ODF 041)	Ekaka (Ateso)	R	Sh	Apply powder topically/make decoction and drink	W	5	No reports
40.* Toona ciliata* M. Roem. (ODF 012)	Yat bwoc/Yat luu pa coo (Luo)	R	T	Pound, mix with water and drink only once.	W	4	No reports
**Moringaceae**							
41. *Moringa oleifera* Lam. (ODF 056)	Moringa*	R/B	T	Decoction	D	2	Bark and root juice used in central Uganda [10].
**Myrtaceae**							
42. *Eucalyptus viminalis* Labill.(ODF 052)	Kalatuc (Luo)	R/L	T	Decoction	D	1	No reports
43. *Eucalyptus* spp. (ODF 053)	Kalatuc (Luo)	L	T	Decoction	D	2	No reports
**Musaceae**							
44.* Musa* spp. (ODF 042)	Amemo (Luo)	L	R	Decoction	D	2	Juice from *Musa balbisiana* and *Musa × paradisiaca* in central Uganda [10].
**Oleaceae**							
45. *Jasminum schimperi* Vatke (ODF 057)	Ederut (Ateso)	R	H	Mix the powder with powder from *C. cyphopetalum* and dissolve in water and drink/apply powder topically	W	10	No reports
**Opiliaceae**							
46. *Opilia amentacea* Roxb. (ODF 054)	Epolokiliok (Ateso)	R	CSh	Apply powder on cuts & also drink	W	1	Root paste is taken internally to cure snakebite by herbalists in India [21].
**Pedaliaceae**							
47.* Sesamum calycinum* subsp. *angustifolium* (Oliv.) Ihlenf. & Seidenst (ODF 015)	Abal/Emelerait (Ateso), Kilode (Luo)	R	H	Crush in water and drink/rub juice on the bitten part	W	2	No reports
**Phyllanthaceae**							
48. *Phyllanthus ovalifolius* Forssk (ODF 043)	Elakas (Ateso)	R	Sh	Powder, mix with cold water and drink 3 times a day for at least 3 days/apply topically	W	3	Root chewed, followed by drinking lots of water to induce vomiting in the management of snakebites in Ethiopia [34]
**Poaceae**							
49.* Imperata cylindrica* (L) Raeusch. (ODF 055)	Obiya (Ateso/Luo)	R	H	Chew	W	1	Root chewed for snakebite in eastern Uganda [10, 11].
50. *Sporobolus pyramidalis* P.Beauv. (ODF 001) (ODF 044)	Ajiki (Luo)	L	H	Decoction	W	1	No reports
**Rubiaceae**							
51. *Gardenia ternifolia* Schumach. & Thonn. (ODF 045)	Ekoroi (Ateso) Odwong (Luo)	R	T	Powder, mix with cold water and drink 3 times a day for at least 3 days/apply topically	W	6	A roots infusion is drunk for snakebite treatment [11].
**Rutaceae**							
*52. Citrus limon* (L.) Osbeck (ODF 047)	Lemun (Luo)	Fr	T	Squeeze out juice and drink	D	1	The juice is drunk [10].
53. *Zanthoxylum chalybeum Engl*. (ODF 048)	Eusuk (Ateso)	S, R		Powder, mix with cold water and drink thrice daily for at least 3 days/apply topically	W	5	No reports
**Sapindaceae**							
54. Zanha golungensis Hiern (ODF 016)	Ekiya Lo’emun (Ateso)	R/St	T	Pound and mix with water and drink twice	W	4	No reports
**Solanaceae**							
55. *Capsicum annuum* L. (ODF 049)	Kamulari (Luo), Emulalu (Ateso)	F	H	Chew/powder, mix with cold water and drink 3 times a day for at least 3 days/apply topically	D	5	No reports
56. Solanum giganteum Jacq. (ODF 017)	Ocok (Luo)	R/L	Sh	Drink ½ cup of decoction/Apply powder to small incisions made around the bite area/burn dry leaves and make victim inhale for severe cases and emergencies	W	2	No reports
57. Solanum incanum L. (ODF 056)	Ocok (Luo)	S	R	Decoction	W	3	Snakebite treatment in Lira district, northern Uganda [35] and Mukono in central Uganda [10].
**Vitaceae**							
*58. Cyphostemma adenocaule* (Steud. ex A.Rich.) Desc. ex Wild & R.B.Drumm. (ODF 058)	Anuno (Luo)	R	H	Decoction	W	5	No reports
59.* Cyphostemma cyphopetalum* (Fresen.) Desc. ex Wild & R.B.Drumm (ODF 034)	Anona (Kumam)	R	H	Pound and squeeze out juice and taken orally	W	10	No reports
**Zingiberaceae**							
60. *Aframomum mildbraedii* Loes. (ODF 018)	Acaet/Asawot (Ateso), Oceyo (Kumam), Ocayo (Luo)	Rh	H	Root juice is drink/powdered & mixed with *C. cyphopetalum* powder & water then drink/applied topically.	W	11	No reports

*H* herb, *Sh* shrub, *T* tree, *Csh* creeping shrub, *Hb* habit, *PU-* parts used, *L* leaves, *R* root, *B* Blb, *S* stem bark, *Sp* sap, *F* fruit, *Bb* bulb, *WD* wild/domesticated, *FM* frequency of mention

*Local name adapted from the English name

The most commonly mentioned plant species were *Steganotaenia araliaceae* (16), *Microglossa pyrifolia* and *Gladiolus dalenii* both at 13; *Aframomum mildbraedii* (11); *Jasminum schimperi*, *Cyathula uncinulata*, *Crinum macowanii* and *Cyphostemma cyphopetalum* (10); *Annona senegalensis* (9); *Echinops longifolius* (9); *Gloriosa superba* and *Indigofera spicata* (8); and *Tamarindus indica* (7). *S. araliaceae* was mentioned by all the TMP in the Ik community. It was used as first aid and is said to cause immediate vomiting only when used by someone bitten by a venomous snake.

**Fig. 2:**
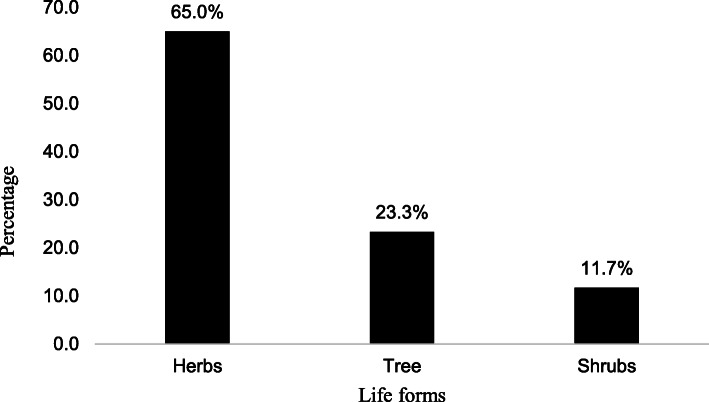
Life forms of medicinal plant species used in the management of snakebites envenomation in Acholi, Teso and Karamoja sub-regions of Uganda

**Fig. 3:**
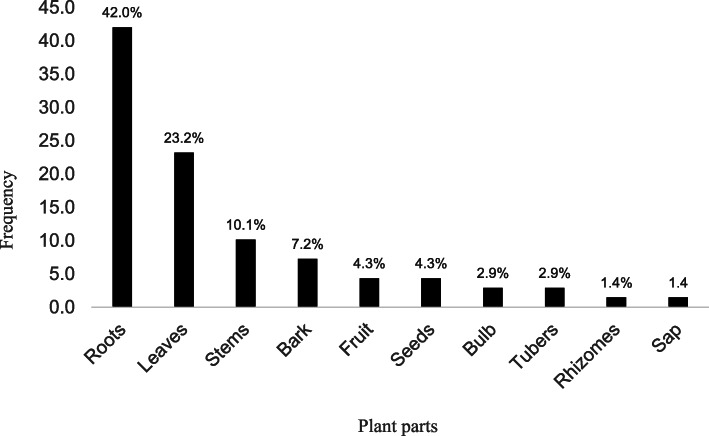
Plant parts used in the management of snakebites envenomation in Acholi, Teso and Karamoja sub-regions of Uganda

Most of the plant species used were herbs (65.0%), followed by trees (23.3%) and shrubs (11.7%) (Fig. 2). The most commonly used plant parts were roots (42.6%), leaves (25.0%), stems (10.3%) and bark (7.4%). The least used parts were rhizomes and sap both at 1.5% (Fig. 3).

### Medicinal plant preparation and administration

**Fig. 4:**
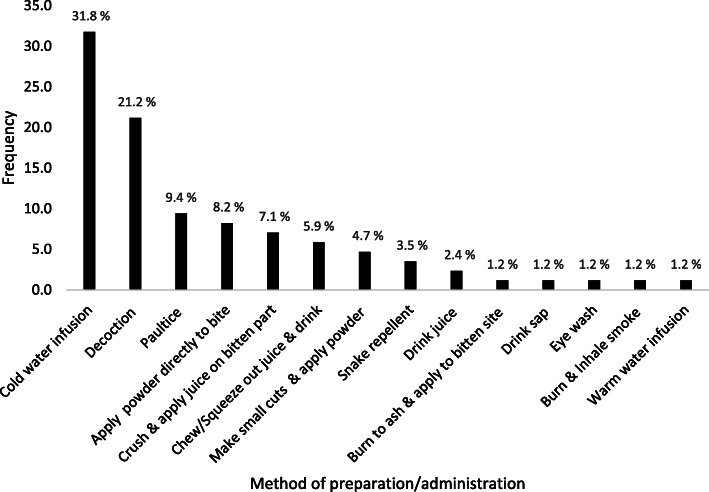
Methods of preparation/administration of the herbal medicines in the Acholi, Teso and Karamoja sub regions of Uganda

The methods of preparation and administration were grouped into thirteen categories. Most of the herbal medicines were prepared for oral administration (62.5%). The rest were administered topically (32.5%) with the exception of inhalation of smoke from burnt plant material (1.2%). The commonest methods of oral application were cold water infusions (31.8%), decoctions (21.2%) and chewing or squeezing juice from the plant material and drinking it (5.9%) (Fig. 4). The commonest topical methods of application were poultices (9.4%) and direct application of powders to the bitten site or juice (8.2%), followed by the application of powder to bite area after making small cuts with a razor blade (4.7%). Some of the plant species were used as snake repellents (3.6) and one specie was used for making an eyewash (1.2%) for cases of ocular envenomation by spitting cobras. One herbalist reported burning the plant material and making the patient inhale the smoke in cases where they were unconscious (1.2%). However, the herbalists were not aware of any specific modes of action of the medicinal plant species they used with the exception of a few species that acted as emetics (Table 2).

In the Ik community in Kaabong district, herdsmen, farmers and hunters usually moved with small quantities of *G. dalenii* powder as a quick remedy in case they were being bitten by a poisonous snake. In case of a snakebite, small cuts are made at the site and the powder applied. Generally, the consensus among the TMP was high in the relatively closed and isolated Ik community. Additionally, the medicinal plant species used by the Ik were generally unique to them and not used by the other communities in Kitgum, Kaberamaido and Serere districts. These included *G. dalenii*, *E. longifolius*, *Cyathula uncinulata* and *Steganotaenia araliaceae.*

### Knowledge acquisition and transfer

Most herbalists acquired their knowledge on the use of medicinal plants for snakebite management from their parents and grandparents (80%) other relatives (12%). Two unique cases (8%) were registered. The first case involved the observation of self-medication in snakes by one herbalist. The said herbalist acquired the knowledge on the use of *Microglossa pyrifolia* for treatment of snakebites by observing a snake wounded in a fight with another snake. The injured snake reportedly recovered from its injuries after ingesting the leaves of *M. pyrifolia*. In the second instance, another herbalist who mainly uses the root of *Opilia amentacea* for all snakebite cases acquired this knowledge from the observation that the stems of *O. amentacea* had whitish scale-like appearance, akin to the scales of some snakes.

### Type of snakebites treated

*Cyathula uncinulata*, *Astripomoe amalvacea*, *Kalanchoe* sp. and *Hoslundia opposita* were specifically mentioned as being used for treating puff adder bites. *Euphorbia hypericifolia* was also used for treating scorpion and spider stings. *Microglossa pyrifolia* was used for treating all types of snakebites except the puff adder bites. *Bryophyllum delagoense* and *Steganotaenia araliaceae* were used for treating cobra bites. In addition, *S. araliaceae* was used as first aid for all snakebites among the Ik only. Most of the plant species used were harvested from the wild (68.75%), whereas 24.24% were domesticated and 6.25% were both domesticated and wild. Thirty-seven (61.6%) of the documented plant species did not have any previous references about use in snakebite treatment literature. However, some of the plant species are from genera with many well-known plant species used for snakebite such as *Solanum*, *Annona*, *Echinops*, *Euphorbia* and *Indigofera*

### Unidentified medicinal plant species used

An additional nine plant species were mentioned by the herbalists for treating snakebite envenomation in Kitgum district (Table 3). However, we were not able to collect voucher specimen for these species for identification for several reasons including wildfires that had destroyed some of their habitats, drought, insecurity near the Uganda Sudan border and difficulty in locating some of the species because they were naturally rare.

**Table 3:** Unidentified plant species used for snakebite treatment

Local name (Acholi)	Part used	Habit	Mode of preparation and administration
1. Obokoleb	T	R/B	Decoction
2. Abangabanga	H	Sd	Chew
3. Lalega dyel	S	R	Decoction
4. Acilo	S	R/L	Decoction
5. Amomo	S	R	Decoction
6. Lacer	S	F/L	Decoction/bath
7. Ngili	H	R	Decoction
8. Te-okwero	S	R	Chew
9. Kokobelle mol	S	L/Sp	Decoction

*T* tree, *H* herb, *S* shrub, *R* root, *B* bark, *L* leaves, *F* flowers, *Sp* sap

## Discussion

Even though most herbs were used singly, some of the herbalists prepared polyherbal formulations for use. One of the TMP disclosed his formula consisting of *Pseudocedrela kotschyi*, *Gardenia ternifolia*, *Zanthoxylum chalybeum*, *Indigofera arrecta* and *Capsicum frutescens*. The herbalists used different herbal formulations to treat their patients. These differences can be attributed to some variations in local flora of the regions, culture, training and the circumstances under which the patients presented. The most frequently cited plant species in this study were selected for farther evaluation of their antivenom potentials in vivo and their phytochemical composition. These experiments are on-going and the findings will be published in due course. The sustainable use of the plant resources raises concerns since most of them are harvested from the wild. To worsen matters, the roots of these plant species are the most widely used parts. Harvesting of roots is destructive to the plant species and is a threat to both the trade of the herbalists and the survival of the plant species. It is encouraging however to note that some herbalists had made attempts to domesticate some of the plant species they used by growing them in their backyards.

Although we reported that most of the plant species recorded in this study do not have any previous documentation for use in snakebite treatment literature, some unique cases stand out. We recorded for the first time the use of *Opilia amentacea* in the treatment of snakebite envenomation in Uganda. Interestingly, the same species is used in India for treating snakebite envenomation [21]. However, *O. amentacea* was only reported by a single renowned traditional healer commonly known as “Dr. Snake”. The use of *O. amentacea* was associated with the doctrine of signatures (DoS) or similarities. The selection of plant species for the treatment of particular conditions because of their resemblance to particular body organs is not a new concept. This DoS or similarities attributes the therapeutic properties of some plants to particular morphological characters or features they possess, i.e. “like treats like” [36]. This particular herbalist began using this plant because of the scale-like and dotted appearance of its bark and its creeping habit. This is the first report on the doctrine of signatures with *O. amentacea* with reference to snakebite. According to Bennett [37], the doctrine of signatures is found throughout the world and has had a long history of use. He further argues that considering the DoS from the classical morphological perspective has rarely led to the discovery of medicinal plants and the approach is therefore unproductive and largely untestable. The DoS cannot therefore be considered scientific [36, 37], although parts of its utility lie in facilitating the process of understanding the subjective, psychological, and spiritual dimensions of nature [38].

Another interesting observation was the routine use of prayers during healing. One particular healer was observed to always begin his plant collection routine in the bush with prayers. He prayed to God beseeching him to give the plant species their healing power before he begun harvesting. He professed the catholic faith and attributed his success to his God. This particular healer had a medicinal plant garden and a special treatment room/hut in which he treated his patients. He got official recognition with a certificate from the ministry of culture in Uganda as early as 1986. The citation of prayers by herbalists who profess Christianity during healing rituals has previously been reported in parts of Uganda such as the west [39]. Prayers form an integral part of the belief system and are believed to make the treatment successful.

The transfer of traditional knowledge is by word of mouth. The TMP identify and train particular children on the identification, preparation and administration of the herbs. We report a unique case of self-medication in snakes. This provides an insight into the potential antivenom properties of *Microglossa pyrifolia*. Although previous studies have reported cases of self-medication especially in primates such as chimpanzees [40, 41], we have not come across previous reports of self-medication in snakes. However, according to Shurkin [42], some lizards are believed to survive venomous snake bites by eating roots of particular plants. It is therefore not farfetched to consider self-medication in snakes.

According to the in-charge of Timu health centre II in Kaabong district, there were relatively many reports of snake bites in the Ik community, but there were few cases reporting to the health facility. Even those who reported to the health centre came several days after being bitten by snakes for supportive treatment after initially managing the snakebites with herbs. The health centre also did not have any antisera for treatment of snake bites.

## Conclusion

TMP widely use several medicinal plant species for treating snakebite envenomation in the post-conflict sub-regions of Acholi, Teso and Karamoja in Uganda. There is a high consensus by herbalists in the Ik community on different plant species used. Most of the plant species are harvested from the wild, prepared as infusions and used orally. The knowledge of medicinal plant use is transmitted orally.

## Methods

### Study design and setting

**Fig. 5:**
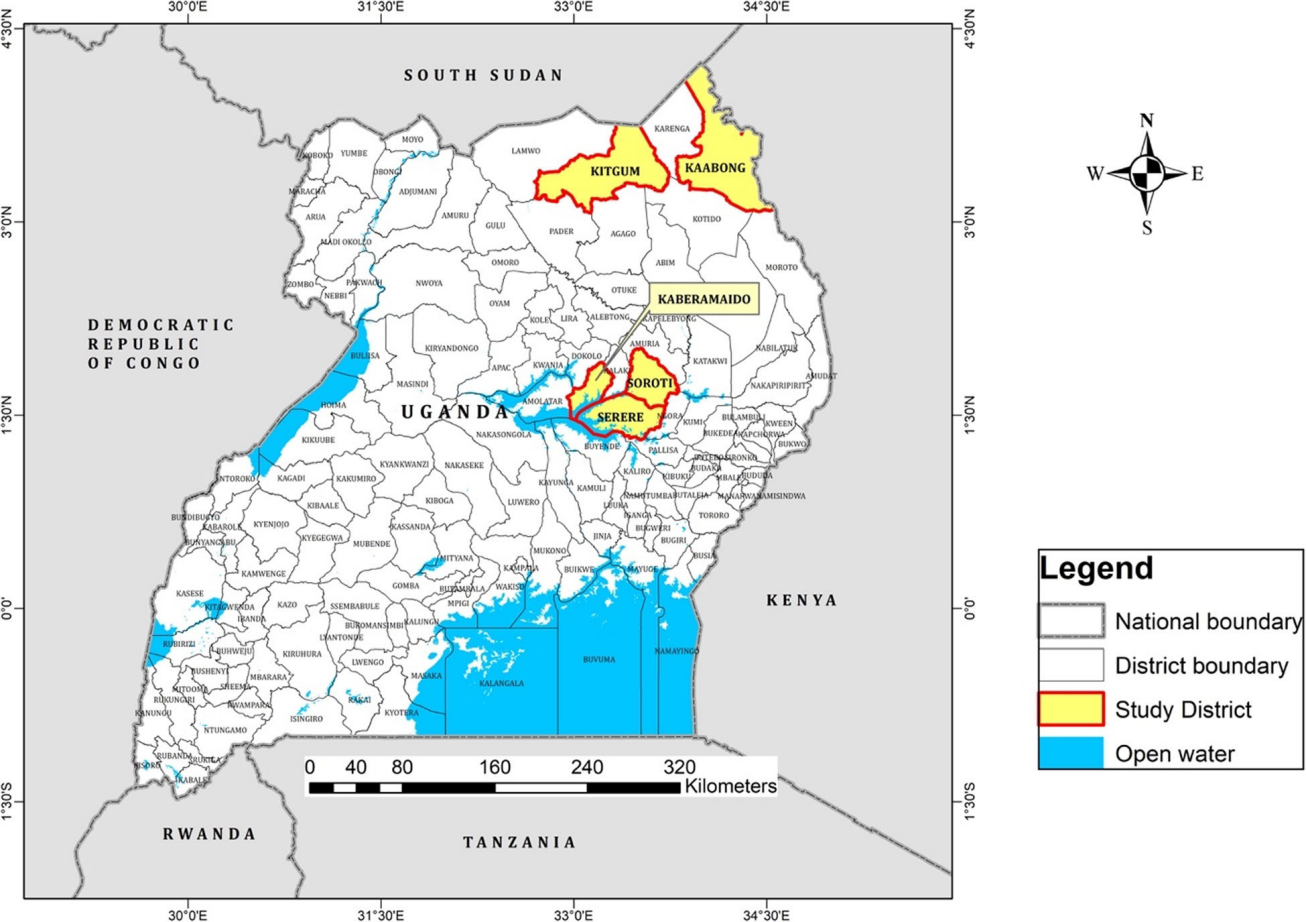
Map of Uganda showing study sites

An ethnopharmacological study was conducted in the districts of Soroti in Mukura/Asuret sub-counties (1.7229° N, 33.5280° E), Serere in n Bugondo, Okulonyo & Kyere sub-county (4994° N, 33.5490° E), Kaberamaido, Anyara sub-county (1.6963° N, 33.2139° E) in the Teso sub-region, Kitgum, Namukora and Orom sub-counties (3.3397° N, 33.1689° E, Acholi sub-region) and Kaabong, Timu sub-county (3.5126° N, 33.9750° E, Karamoja sub-region) (Fig. 5). The data were collected between August and October 2017 using interviews with semi-structured questionnaires These areas have tropical and savanna type vegetation [43]. The study areas were selected because they are recovering from protracted LRA war; they are remote. Additionally, these areas have limited access to modern health facilities with antisera and have been reported to have frequent snakebites [44, 45].

### Characteristics of participants

Traditional medicine practitioners or herbalists with expertise in treating patients bitten by snakes were purposively selected and identified using the snowball technique [46]. In each of the study areas, there are associations of general traditional healers. Each of these associations has specialists such as traditional birth attendants, bone setters and those that treat snakebites within its ranks. Through these networks, we were able to get referrals to the specific snakebite treatment experts.

### Plant collection and identification

Voucher specimens of the plant species mentioned in the study were collected using standard procedures [47] and taken to Makerere University herbarium for identification. The scientific names of the plant species were identified based on the plant list [48]. Plant families were verified using the angiosperm phylogeny group IV [49].

### Data analysis

The data were analysed using simple descriptive statistics in Microsoft Excel 2019.

## Data Availability

Supporting data to this article is publicly available in the Mendeley data repository: Data, V2, 10.17632/g788hgn5t2.2
